# Marine Bacteria as a Source of Antibiotics Against *Staphylococcus aureus*: Natural Compounds, Mechanisms of Action, and Discovery Strategies

**DOI:** 10.3390/md24010044

**Published:** 2026-01-15

**Authors:** Céphas Xuma, Alexandre Bourles, Julien Colot, Linda Guentas, Mariko Matsui

**Affiliations:** 1 Group BIOactivities of NAtural Products and Derivatives (BIONA), Institut Pasteur of New Caledonia, Member of the Pasteur Network, BP 61, 98845 Noumea Cedex, New Caledonia; cxuma@pasteur.nc; 2 Medical and Environmental Bacteriology Group, Institut Pasteur of New Caledonia, Member of the Pasteur Network, BP 61, 98845 Noumea Cedex, New Caledonia; abourles@pasteur.nc (A.B.); julien.colot@cht.nc (J.C.); 3 Gaston-Bourret Territorial Hospital Center of New Caledonia, BP J5, 98 849 Noumea Cedex, New Caledonia; 4 Institut de Sciences Exactes et Appliquées (ISEA), University of New Caledonia, BP R4, 98 851 Noumea Cedex, New Caledonia; 5 Centre for Marine Bioproducts Development, Flinders University, Sturt Road, Health Sciences Building, Bedford Park 5042, South Australia, Australia

**Keywords:** *Staphylococcus aureus*, antibiotic resistance, marine bacteria, bioactive secondary metabolites, marine-derived antibiotics, *Quorum Sensing*, biofilm inhibition

## Abstract

*Staphylococcus aureus* is a major opportunistic pathogen responsible for a wide spectrum of human infections, including severe and difficult-to-treat cases. The emergence of multidrug-resistant strains limits the efficacy of conventional antibiotic therapies and poses a significant global public health challenge. In this context, the search for novel antibiotics has intensified, with increasing interest in marine resources, an ecosystem still largely underexplored. Marine bacteria produce a vast array of secondary metabolites with unique structures and potentially novel modes of antibacterial action. Several compounds isolated from marine bacterial strains have demonstrated promising activity against multidrug-resistant *S. aureus*, including antivirulence effects such as biofilm formation and *Quorum-Sensing* inhibition. This review explores the potential of marine bacteria as a source of new antibiotics against *S. aureus*, discusses both classical and advanced strategies for the discovery of bioactive molecules, and highlights the scientific and technological challenges involved in translating these findings into clinical applications.

## 1. Introduction

### 1.1. Importance of Staphylococcus aureus in Human Infections

*Staphylococcus aureus* is a versatile and ubiquitous human pathogen responsible for a broad spectrum of infections, ranging from superficial wounds and soft tissue infections to life-threatening systemic diseases such as endocarditis, osteomyelitis, and necrotizing pneumonia [1]. Its ability to produce various toxins, including the Toxic Shock Syndrome Toxin-1 (TSST-1), enterotoxins, exfoliatins, and Panton–Valentine Leukocidin (PVL), contributes to severe syndromes such as staphylococcal toxic shock syndrome and scalded skin syndrome, particularly in children and immunocompromised patients [2]. Furthermore, in healthcare settings, *S. aureus* strains are a leading cause of nosocomial infections, especially those associated with medical devices such as catheters, prostheses, or valves, where their capacity to form biofilms complicates eradication [3]. The increasing emergence of resistant strains, especially Methicillin-Resistant *S. aureus* (MRSA), represents a significant challenge in managing these infections. These strains are more resistant to standard treatments. Community-acquired (CA)-MRSA is also responsible for severe infections in the community, even in healthy individuals, which has significantly altered the epidemiology of *S. aureus* infections [4].

### 1.2. S. aureus as a Global Antimicrobial Resistance Threat

Antimicrobial resistance (AMR) represents one of the major global health issues of the 21st century. Indeed, the latest estimation associated more than 4.71 million deaths with AMR in 2021 [5]. Among AMR-pathogenic bacteria, *S. aureus*, particularly MRSA, accounted for the largest increase in associated deaths globally, from 261,000 in 1990 to 550,000 in 2021 [5]. MRSA was also identified as the world’s deadliest antibiotic-resistant pathogen in 2021 [5], further emphasizing the strategic importance of this bacterium in the development of new therapeutic alternatives. Thus, *S. aureus* is regarded as one of the most clinically important multidrug-resistant (MDR) threats globally according to the recent global bacterial priority pathogens list (global BPPL) of antibiotic-resistant bacteria by the World Health Organization (WHO) [6].

### 1.3. The Emergence of Antibiotic Resistance in S. aureus

Interestingly, *S. aureus* was the first bacterium to show clinically detected resistance to antibiotics [7]. As early as 1940, one year before the first clinical use of penicillin, resistance of *S. aureus* to this antibiotic was already reported [7]. To counteract this growing resistance, methicillin, the first semi-synthetic penicillin, was introduced in 1959 to treat infections caused by penicillin-resistant *S. aureus* [8]. However, by 1961, MRSA strains had been detected [9]. Over time, the emergence of MDR variants further worsened the problem, and in 1996, Japan reported the first *S. aureus* strain with reduced susceptibility to vancomycin, called vancomycin-intermediate *S. aureus* (VISA) [10], followed by the identification of the first clinical vancomycin-resistant *S. aureus* (VRSA) strain in the United States in 2002 [11]. Today, VISA and VRSA strains remain a global threat, requiring increasingly potent treatments. Furthermore, the growing diversity and adaptability of resistant *S. aureus* strains make treatment increasingly difficult, particularly in the case of MRSA, even with the introduction of newer anti-MRSA agents such as ceftaroline, ceftobiprole, cefiderocol, and delafloxacin [12,13,14,15]; early reports of decreased susceptibility or resistance to these agents underscore the ongoing need for vigilant surveillance, antimicrobial stewardship, and the development of novel therapies. For example, linezolid resistance mechanisms (mutations in 23S rRNA, acquisition of the *cfr* gene) have been documented in *S. aureus* [16]. The alarming pattern is that each time a new class or agent is deployed, *S. aureus* often finds a way to adapt.

### 1.4. Mechanisms of Resistance of S. aureus

**Figure 1:**
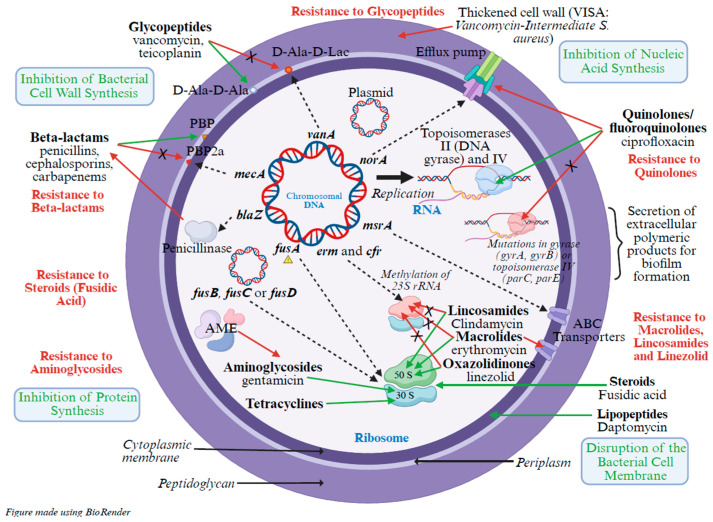
Mechanisms of antibiotic and resistance in *S. aureus.* This figure summarizes key antibiotic targets (green arrows) and associated resistance mechanisms (red arrows) in *S. aureus*. β-lactam resistance that involves the *mecA* gene encoding PBP2a and *blaZ*-encoded β-lactamase (e.g., Penicillinase). Glycopeptide resistance includes *vanA*-mediated d-Ala-d-Lac substitution and cell wall thickening in VISA strains. Aminoglycoside resistance arises from enzymatic modification. Fluoroquinolone resistance involves *norA*-encoded efflux pumps and mutations in *gyrA*, *gyrB*, *parC*, and *parE*. Macrolide, lincosamide, and oxazolidinone resistance is mediated by methylation of 23S rRNA by *erm* and *cfr* genes, and by *msrA*-mediated ABC transporters. Fusidic acid targets the elongation factor G (EF-G) involved in protein synthesis. Resistance occurs either via chromosomal mutations in *fusA*, which reduce fusidic acid binding, or through acquisition of plasmid-borne *fusB*, *fusC*, or *fusD* genes encoding proteins that protect EF-G from the antibiotic’s action. Biofilm formation contributes to multidrug resistance by enhancing persistence, gene transfer, and protection from antibiotics. Abbreviations: MRSA = Methicillin-Resistant *S. aureus*; VISA = Vancomycin-Intermediate *S. aureus*; PBP = Penicillin-Binding Protein; AME = Aminoglycoside-Modifying Enzyme; ABC transporters = ATP-Binding Cassette transporters. Created with BioRender.com. C, Xuma (2026).

*S. aureus* displays a remarkable arsenal of resistance mechanisms, both genetically encoded on the chromosome and acquired via horizontal gene transfer. These include enzymatic antibiotic degradation, target site modification, efflux pumps, and biofilm formation. The diversity and the association of these mechanisms significantly contribute to treatment failure and persistent infections (Figure 1). In addition to these intrinsic and acquired mechanisms, co-selection of resistance mechanisms linked to the widespread use of topical antibiotics, such as creams containing fusidic acid [17,18], also significantly contributes to the emergence of resistance, particularly in community settings. Furthermore, several *S. aureus* strains described in the literature harbor multiple resistance mechanisms simultaneously. This highlights the importance of distinguishing MRSA strains that remain sensitive to certain antibiotic classes from those that are truly multidrug-resistant [19]. This distinction is clinically relevant. An MRSA strain still susceptible to other antibiotics may not pose a major therapeutic challenge. In contrast, MRSA strains with additional resistances represent a much more serious threat. Similarly, methicillin-sensitive *S. aureus* (MSSA) infections are generally treatable, but in patients allergic to β-lactams [20], MSSA strains resistant to other antibiotic classes present significant clinical management difficulties.

#### 1.4.1. Resistance to β-Lactam Antibiotics

Resistance to β-lactam antibiotics is primarily mediated by two major mechanisms: enzymatic hydrolysis of the β-lactam ring and modification of penicillin-binding proteins (PBPs), rendering the drug ineffective. The *blaZ* gene encodes a β-lactamase (penicillinase) that hydrolyzes penicillin G, providing low-level resistance to penicillin in *S. aureus* [21]. In contrast, the *mecA* gene encodes PBP2a, a transpeptidase with low affinity for β-lactams, allowing continued peptidoglycan synthesis in the presence of these antibiotics. This is the hallmark of the MRSA phenotype [22].

#### 1.4.2. Resistance to Glycopeptides

Resistance to vancomycin and other glycopeptides can result from either acquisition of resistance genes or structural alterations in the cell wall. The *vanA* gene, typically acquired from enterococci, modifies peptidoglycan precursors by replacing the D-Ala-d-Ala terminus with d-Ala-d-Lac, significantly reducing vancomycin binding affinity [23]. In vancomycin-intermediate *S. aureus* (VISA), resistance emerges through stepwise mutations that lead to thickening of the cell wall, trapping vancomycin molecules and impeding their access to the target [24].

#### 1.4.3. Resistance to Aminoglycosides

Resistance to aminoglycosides is mainly mediated by enzymatic modification of the antibiotic molecule, preventing its binding to the bacterial ribosome. Three families of aminoglycoside-modifying enzymes (AMEs) are involved: acetyltransferases (AAC), nucleotidyltransferases (ANT), and phosphotransferases (APH). These enzymes have been proven to be capable of chemically modifying aminoglycosides, thereby rendering them inactive [25,26,27].

#### 1.4.4. Resistance to Quinolones

Quinolone resistance arises from both active efflux and target site modification. The *norA* gene encodes a proton-motive force-dependent efflux pump, reducing intracellular fluoroquinolone concentrations and conferring low-level resistance [28]. Higher-level resistance is associated with mutations in DNA gyrase (*gyrA*, *gyrB*) and topoisomerase IV (*parC*, *parE*), which reduce antibiotic binding to these essential enzymes [28,29].

#### 1.4.5. Resistance to Macrolides, Lincosamides, and Linezolid

Resistance to this group of antibiotics involves ribosomal target modification and active efflux mechanisms. *erm* and *cfr* genes encode methyltransferases that methylate the 23S rRNA of the 50S ribosomal subunit, blocking antibiotic binding and leading to cross-resistance across the MLS_B group (macrolides, lincosamides, streptogramin B) and to linezolid [16]. Additionally, the *msrA* gene encodes an ABC transporter, promoting active efflux of macrolides and streptogramin B [30].

#### 1.4.6. Resistance to Steroids (Fusidic Acid)

Fusidic acid, a steroidal antibiotic, inhibits bacterial protein synthesis by targeting elongation factor G (EF-G). Resistance to fusidic acid in *S. aureus* arises through two main mechanisms: (1) chromosomal mutations in the *fusA* gene, which encodes EF-G, reduce fusidic acid binding affinity, and (2) acquisition of plasmid-borne resistance genes (*fusB*, *fusC*, or *fusD*), which encode proteins that protect EF-G from fusidic acid inhibition [31]. These mechanisms significantly compromise the clinical efficacy of fusidic acid, especially when used topically in monotherapy, which promotes resistance selection [17]. Fusidic acid resistance is of particular concern in community settings due to the frequent use of fusidic acid-containing creams and ointments.

#### 1.4.7. Biofilm Formation

Biofilms contribute to resistance by acting as a physical and metabolic barrier to antibiotics and immune responses. *S. aureus* forms organized biofilm structures embedded in an extracellular matrix composed primarily of proteins, polysaccharides, and extracellular DNA [32], which limits antibiotic penetration, promotes horizontal gene transfer, and harbors dormant persistent cells. Biofilms are particularly problematic in device-related infections, where eradication often requires both antibiotic therapy and device removal [33].

### 1.5. Therapeutic Challenges and Economic Burden of Treating S. aureus Infections

Although MSSA is generally more treatable than MRSA, certain MSSA strains display multidrug resistance (MDR), including resistance to macrolides (Erythromycin: 50.6% resistance) and lincosamides (Clindamycin: 44.5% resistance), complicating clinical management [34]. These MDR MSSA profiles, often overlooked, underscore the fact that methicillin susceptibility alone does not guarantee favorable therapeutic outcomes, particularly in deep-seated or chronic infections [34,35]. Moreover, MSSA continues to cause a significant proportion of *S. aureus* infections globally. As such, the eradication of MRSA would not in itself eliminate the broader clinical burden associated with *S. aureus* [35]. MRSA adds further complexity due to its resistance to multiple antibiotic classes, including first-line β-lactams and second-line intravenous agents like glycopeptides, which are reserved for severe infections [36,37]. Vancomycin, while still widely used, suffers from limited tissue penetration, suboptimal efficacy in bacteremia and bone infections, and nephrotoxicity, especially with prolonged use [38]. The emergence of VISA and VRSA strains further limits treatment options and has led to increased reliance on alternatives such as linezolid and daptomycin [39]. However, these agents are associated with hematologic, neurologic, or muscular toxicities, which restrict their use in some clinical situations [40,41]. In addition, many antibiotics fail to reach effective concentrations in intracellular compartments or avascular tissues, limiting their activity in infections such as osteomyelitis or device-associated infections. *S. aureus*’s ability to form biofilms on surfaces such as catheters, prosthetic joints, and heart valves further impairs antibiotic penetration and promotes persistence, frequently requiring device removal in addition to prolonged treatment [42].

The therapeutic limitations of current antibiotics translate into increased healthcare costs and longer hospital stays. For instance, MRSA infections have been shown to increase per-patient hospital expenditures by up to USD 9606 in China [43]. In Canada, the cost of managing antimicrobial-resistant infections, including those caused by MRSA, reached approximately USD 272 million in 2019 [44]. Furthermore, microbiological confirmation of MRSA can take several days, delaying the administration of effective, targeted therapy [45]. This delay contributes to poor clinical outcomes, especially in severe infections such as bacteremia and pneumonia [46]. Together, these factors highlight the pressing need for novel antibacterial strategies, particularly agents with improved tissue penetration, activity against biofilms, and reduced toxicity profiles, to address the persistent threat of *S. aureus* resistance.

### 1.6. Objectives and Methodology

This review aims to explore Marine Natural Products (MNPs) derived from marine bacteria and their potential as novel antibiotics against *S. aureus*. To achieve this, a systematic literature search was conducted using the Web of Science (WOS) [47] and MarinLit [48] databases. Keywords were combined using Boolean operators (AND, OR) to comprehensively cover topics related to marine bacteria, secondary metabolites, and antibacterial activity targeting *S. aureus* and its resistant strains, including MRSA. Inclusion criteria focused on scientific relevance, availability of structural and biological data on compounds, and chemical novelty. The research covered publications from 1900 to August 2025. Key terms included phrases such as “marine bacteria,” “marine-derived metabolites,” “antibacterial activity,” “anti-*Staphylococcus aureus*,” and “anti-MRSA,” along with related synonyms. The search also targeted various marine habitats (e.g., sediments, sponges, deep-sea environments) to ensure a broad representation of bacterial sources.

## 2. Antibacterial Potential of Marine Bacteria Compounds Against *S. aureus*

### 2.1. Interest in Marine Resources as a Reservoir of New Bioactive Molecules

Natural resources have historically been a prolific source of bioactive compounds for drug discovery, with natural products accounting for an average of 32 ± 9% of all approved therapeutic agents between 1981 and 2019 [49]. Among these, the marine environment stands out as an exceptionally rich yet underexploited reservoir of novel bioactive molecules produced by diverse organisms, including sponges, algae, corals, and especially microorganisms [50,51,52]. The extreme and variable conditions characteristic of marine habitats, such as high hydrostatic pressure in the deep sea, intense salinity fluctuations, and fierce interspecies competition, have driven marine organisms to evolve unique biochemical adaptations [53,54,55]. These adaptations often manifest with the synthesis of structurally novel secondary metabolites with broad biological activities, including antibacterial, antifungal, antiviral, anti-inflammatory, and anticancer properties [56,57,58,59]. Notably, several marine-derived compounds have demonstrated potent antimicrobial activity against *S. aureus*, including multi-drug-resistant strains [60,61], highlighting the therapeutic potential of this environment.

### 2.2. Marine Bacteria as a Source of New Antibiotics

Historically, the discovery of MNPs focused primarily on macro-organisms such as sponges and cnidarians. Between 1985 and 2012, approximately 75% of isolated MNPs with reported bioactivities originated from invertebrates, including cnidarians, of which about 57% demonstrated significant biological activities [62]. However, recent advances have revealed the pivotal role of marine microorganisms, particularly bacteria, in the biosynthesis of many of these compounds [63]. These microbes often exist in close symbiotic relationships with their hosts, providing chemical defenses against pathogens while benefiting from a stable habitat and nutrient supply [64,65,66,67,68,69]. This mutualism underscores the ecological importance of microbial interactions in marine environments and has prompted intensified exploration of both planktonic (water column) and benthic (sediment) niches for novel microbial strains [70].

Marine bacteria exhibit remarkable genetic and metabolic diversity, producing a wide array of secondary metabolites with unique structural and functional properties [71,72]. Notably, taxa such as Actinobacteria (e.g., *Streptomyces* spp.) and Firmicutes (particularly *Bacillus* spp.) dominate the spectrum of marine bacterial producers, each evolving distinct biochemical strategies adapted to marine conditions [73,74]. These bacteria biosynthesize diverse compound classes, including polyketides, non-ribosomal peptides, terpenes, and alkaloids, many of which show promising antimicrobial properties against *S. aureus* resistant pathogens (see Table 1).

While Firmicutes, especially *Bacillus* species, have historically been the most studied bacteria for their antimicrobial metabolites, largely due to ease of isolation and cultivation, marine Actinobacteria remain less explored because of their stringent growth requirements and slow growth rates *in vitro* [75,76,77]. Nevertheless, recent research has highlighted the rich biosynthetic potential of marine Actinobacteria, characterized by extensive gene clusters encoding polyketide synthases (PKS) and non-ribosomal peptide synthetases (NRPS), which generate structurally complex and biologically potent secondary metabolites [78]. This growing interest reflects the untapped potential of these bacteria as a critical source of novel antibiotics in the ongoing fight against antimicrobial resistance.

**Table 1:** Marine-derived natural compounds with antimicrobial activity against *S. aureus* strains (published between 2000 and 2025). Compounds are categorized by chemical class. The table includes the compound classes, names, marine source organisms, targeted bacterial strains, antimicrobial activity [reported as minimal inhibitory concentration (MIC), 50% inhibitory concentration (IC_50_), or zone of inhibition (ZI)], known mechanisms of action (MOA), and corresponding references. Antibacterial activities are expressed in µg/mL, µM, or inhibition zone diameters (mm), depending on the available data. The environmental source of the producing microorganisms is indicated in parentheses (e.g., sediment, sponge, algae, etc.).

Class	Subclass	Marine Bacterial Strain (Source)	Compound	*S. aureus* Strains Tested	MIC, IC_50_ or ZI	MOA	References
Alkaloids	-	*Marinispora* sp. NPS12745 (sediment)	Lynamicin A	MSSA (ATCC29213) MRSA (ATCC43300)	MIC = 4.3 (MSSA), 4.7 (MRSA) µg/mL	-	[79]
	-	*Marinispora* sp. NPS12745 (sediment)	Lynamicin B	MSSA (ATCC29213) MRSA (ATCC43300)	MIC = 1.8 (MSSA), 2.2 (MRSA) µg/mL	-	[79]
	-	*Marinispora* sp. NPS12745 (sediment)	Lynamicin C	MSSA (ATCC29213) MRSA (ATCC43300)	MIC = 2.5 (MSSA), 3.5 (MRSA) µg/mL	-	[79]
	-	*Marinispora* sp. NPS12745 (sediment)	Lynamicin D	MSSA (ATCC29213) MRSA (ATCC43300)	MIC = 6.2 (MSSA), 6.2 (MRSA) µg/mL	-	[79]
	-	*Marinispora* sp. NPS12745 (sediment)	Lynamicin E	MSSA (ATCC29213) MRSA (ATCC43300)	MIC = > 45 (MSSA), 27 (MRSA) µg/mL	-	[79]
	-	*Micromonospora* sp. WMMA-2495 (tunicate)	Phallusialides A and B	MRSA (ATCC33591)	MIC = 32 µg/mL	-	[80]
	-	*Pseudomonas aeruginosa* (eal fish)	1-methyl-1,4 dihydroquinoline	MSSA MRSA	MIC = 50–75 µg/mL	-	[81]
	-	*Pseudomonas* sp. UJ-6 (seawater)	1-acetyl-beta-carboline	MRSA (KCCM40510, KCCM40511) MRSA clinical isolates	MIC = 32 (KCCM40510), 64 (KCCM40511) µg/mL	-	[82]
	-	*Streptomyces* sp. CNQ-418 (sediment)	Marinopyrrole A	MSSA (NCTC8325) MRSA	MIC = 0.25 µg/mL (MSSA) MIC_90_ = 0.31 µg/mL (MRSA)	Membrane depolarization and dissipation of the proton motive force (PMF) essential for cell viability	[83,84]
	-	*Streptomyces* sp. CNQ-418 (sediment)	Marinopyrrole B	MRSA	MIC_90_ = 0.63 μg/mL		[84]
	-	*Streptomyces* sp. CNQ-418 (sediment)	Marinopyrrole C	MRSA	MIC_90_ = 0.16 μg/mL		[83]
	-	*Streptomyces* sp. CNQ-418 (sediment)	Marinopyrrole F	MRSA	MIC_90_ = 3.1 μg/mL		[83]
	-	*Streptomyces* sp. HZP-2216E (seaweed)	Streptopertusacin A	MRSA (ATCC43300)	MIC = 40 µg/mL	-	[85]
	-	*Streptomyces* sp. SCSIO 11791 (deep-sea sediment)	Dionemycin	MSSA (ATCC29213) MRSA clinical isolates	MIC = 1 (MSSA), 2 (MRSA) µg/mL	-	[86]
	-	*Streptomyces* sp. SCSIO 11791 (deep-sea sediment)	6-OMe-7′,7″-dichorochromopyrrolic acid	MSSA (ATCC29213) MRSA clinical isolates	MIC = 3 (MSSA), 32–128 (MRSA) µg/mL	-	[86]
	-	*Streptomyces* sp. ZZ1118 (shrimp)	Streptoindole A	MRSA (ATCC43300)	MIC = 25 µg/mL	-	[87]
	-	*Streptomyces* sp. ZZ1118 (shrimp)	Streptoindole B	MRSA (ATCC43300)	MIC = 7 µg/mL	-	[87]
	-	*Streptomyces* sp. ZZ1118 (shrimp)	Streptoindole D	MRSA (ATCC43300)	MIC = 25 µg/mL	-	[87]
	-	*Streptomyces* sp. ZZ741 (marine mud)	Streptoglutarimides A–J	MRSA (ATCC43300)	MIC = 9–11 μg/mL	-	[88]
	-	*Streptomycetes* sp. SMS636 (sediment)	Lansai D, E and F, imidazo[4,5-*e*]-1,2,4-triazine	MSSA (ATCC6538) MRSA (ATCC29213)	MIC = > 100 µg/mL		[89]
	-	*Streptomycetes* sp. SMS636 (sediment)	1-*N*-methyl-(E, Z)-albonoursin	MSSA (ATCC6538) MRSA (ATCC29213)	MIC = 12.5 µg/mL (MSSA) MIC = 25 µg/mL (MRSA)	Importance of the (E, Z) configuration of the double bond for activity	[89]
	-	*Streptomycetes* sp. SMS636 (sediment)	Streptonigrin	MSSA (ATCC6538) MRSA (ATCC29213)	MIC = 0.78 µg/mL (MSSA, MRSA)	Cause oxidative damage and lethal bacterial DNA strand breakage	[89,90]
	-	*Vibrio ruber* ZXR-93 (seawater)	Vibripyrrolidine A	MSSA (ATCC6538)	MIC = 1.95 µg/mL	-	[91]
	-	*Vibrio ruber* ZXR-93 (seawater)	Vibridiazinane A	MSSA (ATCC6538)	MIC = 0.98 µg/mL	-	[91]
	-	*Vibrio ruber* ZXR-93 (seawater)	Vibridiazinane B	MSSA (ATCC6538)	MIC = 3.90 µg/mL	-	[91]
	-	*Verrucosispora* sp. strain MS100047 (deep-sea sediment)	Glycerol 1-hydroxy-2,5-dimethyl benzoate	MRSA	MIC = 12.5 µg/mL	-	[92]
Bromophenol derivative	-	*Nocardiopsis* sp. SCA21 (sediment)	4-bromophenol	MSSA (ATCC12600) MRSA (ATCC NR-46117)	MIC = 62.5 (MSSA), 15.62 (MRSA) µg/mL	-	[93]
Fatty acid derivatives	-	*Aequorivita* sp. (sediment)	*R*-(+)-*N*-[15-methyl-3-(12-methyltridecanoyloxy)-hexadecanoyl] glycine and N-terminal glycine unit	MRSA (clinical isolate DSM18827)	IC_50_ = 22–145 μg/mL	-	[94]
Halogenated aromatic compounds and aromatic compounds	Biphenyl carboxylic acid	*Pseudoalteromonas phenolica* O-BC30^T^	MC21-B (2,2′,3-tribromo-biphenyl-4-4′-dicarboxylic acid)	MSSA (ATCC25923) MRSA (ATCC33591)	MIC = 1–4 µg/mL	-	[95]
	Biphenyl diol	*Actinomadura* sp. DS-MS-114 (deep-sea sediment)	5,6-dihydro-1,8-dihydroxy-3-methylbenz[*a*]anthracene-7,12-quinone	MSSA (NBRC12732)	ZI = 12.7 mm (100 µg of 50 µL at 2 mg/mL)	-	[96]
		Actinomycete CNQ-525 (sediment)	Chlorinated dihydroquinones	MRSA	MIC = 1.90–15.6 μg/mL	-	[97]
		*Nocardiopsis* sp. HDN154086 (sediment)	Nocarterphenyl D	MRSA	MIC > 50 µM	-	[98]
		*Nocardiopsis* sp. HDN154086 (sediment)	Nocarterphenyls F	MRSA	MIC = 6.2 µM	-	[98]
		*Pseudoalteromonas luteoviolacea* (seaweed)	2, 4-dibromo-6-chlorophenol	MRSA 9551	ZI = 20 mm (1.0 mg/mL, 40 µL)	-	[99]
		*Pseudoalteromonas phenolica* O-BC30^T^	MC21-A (3,3′,5,5′-tetrabromo-2,2′-biphenyldiol)	MSSA (ATCC25923) MRSA (ATCC33591)	MIC = 1–2 µg/mL	-	[100]
		*Salinispora arenicola* BRA-213 (sediment)	Salinaphthoquinones B and D	MSSA (ATCC29213) MRSA (ATCC43300)	MIC = 31–125 µg/mL	-	[101]
		*Streptomyces* sp. 1425S.R.1a.1 (mollusk)	7,8-dideoxygriseorhodin C	MRSA (ATCC43300)	MIC = 0.08–0.12 µg/mL	-	[102]
	Biphenyl diol	*Streptomyces* sp. CNQ-418 (sediment)	4,4′,5,5′-Tetrachloro-1′H-1,3′-bipyrrole-2,2′-diyl)bis((2-acetoxyphenyl)methanone)(1), 4,4′,5′-Trichloro-5-methoxy-1′H-1,3′-bipyrrole-2,2′-diyl)bis((2-hydroxyphenyl)methanone (2), N-(2-(3,4′,5′-Trichloro-2′,5-bis(2-hydroxybenzoyl)-1′H-1,3′-bipyrrol-2-ylthio)ethyl)acetamide (3) and 4,4′,5′-Trichloro-5-(dimethylamino)-1′H-1,3′-bipyrrole-2,2′-diyl)bis((2-hydroxyphenyl) methanone (4)	MRSA	MIC_90_ = 0.78–6.3 μg/mL	-	[83]
	Biphenyl diol	*Streptomyces* sp. EG1 (sediment)	Mersaquinone	MRSA (TCH1516)	MIC = 3.36 μg/mL	-	[103]
	Biphenyl diol	*Streptomyces* sp. EG32 (sediment)	Chlororesistoflavins A and B	MRSA	MIC = 0.25–2 µg/mL	-	[104]
	Biphenyl diol	*Streptomyces* sp. strain CA-271078 (ascidian)	3-chloro-6,8-dihydroxy-8-α-lapachone	MRSA (MB5393)	MIC > 64 µg/mL	-	[105]
Hybrids	Peptide-polyketide	*Marinispora* sp. NPS008920 (sediment)	Lipoxazolidinones A–C	MSSA (ATCC29213) MRSA (ATCC43300)	MIC = 1.0–6.0 μg/mL	-	[106]
		*Pseudomonas aeruginosa* 1682U.R.0a.27 (shipworm)	Mindapyrroles A–C	MSSA (ATCC29213) MRSA (ATCC43300)	MIC = 4 to >32 µg/mL	-	[107]
		*Streptomyces microflavus* MBTI36 (sediment)	Chromomycins A_9_, Ap, A_2_ and A_3_	MSSA (ATCC25923, CCARM0027, CCARM0204, CCARM0205 and CCARM3640) and MRSA (CCARM3089, CCARM3090, CCARM3634, CCARM3635, ATCC43300, ATCC700787 and ATCC700788)	MIC = 0.06–0.25 μg/mL	RNA polymerase inhibition by chromomycin A_3_	[108,109]
		*Streptomyces* sp. ZZ820 (coastal soil)	Chromomycin A_3_	MRSA (ATCC43300)	MIC = 0.59 µM	RNA polymerase inhibition by chromomycin A_3_	[109,110]
		*Verrucosispora* sp. strain MS100047 (deep-sea sediment)	Proximicin B	MSSA (ATCC29523) MRSA (EMRSA-15, EMRSA-16) Mutidrug resistant MDR *S. aureus* (SA1199B)	MIC = 8 (MSSA), 4–8 (MRSA), 4 (MDR) µg/mL	-	[92,111]
	Chalcone-terpenoid	*Streptomyces* sp. G246 (sponge)	5′-lavandulyl-4′-methoxy-2,4,2′,6′-tetrahydroxylchalcone	MSSA (ATCC25923)	MIC = 1 µg/mL	-	[112]
	Flavonoid-terpenoid	*Streptomyces* sp. G246 (sponge)	6-lavandulyl-7-methoxy-5,2′,4′-trihydroxylflavanone	MSSA (ATCC25923)	MIC = 32 µg/mL	-	[112]
	Phenazine-terpene	Actinomycete strains CNS-284 and CNY-960 (sediment)	Marinocyanins A–F	MSSA	MIC = 2.37–36.62 µM	-	[113]
	Terpene-polyketide	*Streptomyces* sp. CA-271078	Napyradiomycin analogs	MRSA (MB5393)	MIC = 3 to >96 µg/mL	-	[114]
		*Streptomyces* sp. strain CA-271078 (ascidian)	3-hydroxy-10a-(3-chloro-6-hydroxy-2,2,6-trimethylcyclohexylmethyl)-6,8-dihydroxy-2,2-dimethyl-3,10a-dihydro-2H-benzo[g]chromene-5,10-dione (1), 4-dehydro-4a-dechloronapyradiomycin A1 (2), napyradiomycin A1 (3)	MRSA (MB5393)	MIC = 0.5 to >64 µg/mL	-	[105]
Peptides	-	*Bacillus pumilus* SF214	Pumilacidin	MSSA	-	Alteration in motility and biofilm formation	[115,116]
	-	*Bacillus* sp. (Tube worm)	Bogorol A	MRSA	MIC = 2 µg/mL	Disruption of bacterial cell membrane leading to cell lysis	[117,118]
	-	*Bacillus subtilis* 109GGC020 (sediment)	Bacilotetrins A and B	MRSA (ATCC25923), XU212, SA1199B and RN4220)	MIC = 8–32 µg/mL	-	[119]
	-	*Paenibacillus profundus* sp. nov. SL 79 (sediment)	Glyceryl-D-leucyl-D-alanyl-D-leucyl-D-leucyl-L-valyl-D-leucylD-alanine	MSSA (CIP65.8T)	ZI = 24 mm (1 mg/mL)	-	[120]
	-	*Saccharomonospora* sp. CNQ-490	Taromycins A and B	MRSA (A8819-DapS, A8817-DapR and 0325)	MIC = 3.1–50 µg/mL	-	[121]
	-	*Streptomyces* sp. CNS-575 (sediment)	Fijimycins A–C (1–3), Etamycin A (4)	MRSA (ATCC33591, Sanger 252, UAMS1182)	MIC_100_ = 4 à > 32 μg/mL	Inhibition of bacterial protein synthesis by targeting the ribosome (3 and 4)	[122,123]
	-	*Streptomyces* sp. IMB094 (sediment)	Neo-actinomycin A, actinomycins D and X_2_	MSSA (ATCC29213, isolates) MRSA (ATCC33591, isolates)	MIC = 0.125–64 µg/mL	Intercalates DNA blocking transcription (RNA polymerase) and DNA replication by Actinomycin D	[124]
	-	*Streptomyces* sp. LHW52447 (sponge)	Actinomycins D_1_–D_4_ and actinomycin D	MRSA (P172, P172 and ATCC 33591)	MIC = 0.125–1 μg/mL	Intercalates DNA blocking transcription (RNA polymerase) and DNA replication by Actinomycin D	[125]
	-	*Streptomyces* sp. LS298 (sponge)	Quinomycin G	MSSA (ATCC29213, isolates) MRSA (ATCC33591, isolates)	MIC = 32 µg/mL	-	[126]
	-	*Streptomyces* sp. ZZ338 (sea squirts)	Actinomycin D, V and X_0β_	MRSA (ATCC43300)	MIC = 0.08–0.61 µM	Intercalates DNA blocking transcription (RNA polymerase) and DNA replication by Actinomycin D	[127]
Phenazine derivatives	-	Actinomycete strains CNS-284 and CNY-960 (sediment)	2-bromo-1-hydroxyphenazine (1) and lavanducyanin (2)	MSSA	MIC = 2.92 (2) and 56.93 µM (1)	-	[113]
Phthalate ester	-	*Nocardiopsis* sp. SCA21 (sediment)	Bis (2-ethylhexyl) phthalate	MSSA (ATCC12600)	MIC = 125 µg/mL	-	[93]
Polyketides	-	*Bacillus amyloliquefaciens* MTCC 12716 (red algae)	Methyl 1′-((2E,4E,14E)-9,12-dihydroxy-15-isopropyl-1,6-dioxohexadecahydro [1] oxacyclononadecino[3,4-f] isobenzofuranyl) benzoate (1), (E)-Ethyl 15-ethyl-9,12-dihydroxy-25-(2′-hydroxy-3′-(methoxycarbonyl)phenyl)-1-oxo-octadecahydro-1*H*-furopyrano[2,3-c]oxacyclononadecine-6-carboxylate (2) and (E)-Ethyl 15-ethyl-12-hydroxy-25-(2′-hydroxy-3′-(methoxycarbonyl)phenyl)-24-methyl-1-oxo-icosahydro-1*H*-furopyrano[2,3-c]oxacyclononadecine-6-carboxylate (3)	MRSA (ATCC33592)	MIC = 21.00 ± 0.05–35.00 ± 0.01 µg/mL	Targeting bacterial deformylase	[128]
	-	*Micromonospora harpali* SCSIO GJ089 (sediment)	Microsporanates A, B and tetrocarcins A, B	MSSA (ATCC29213) MRSA (clinical isolate shhs-A1)	MIC = 32 µg/mL	-	[129]
	-	*Micromonospora* sp. CA-214671 (sediment)	Phocoenamicins B and C	MRSA (MB5393)	MIC = 8–64 µg/mL	-	[130]
	-	*Micromonospora* sp. RJA4480 (sediment)	3-amino-27-demethoxy-27-hydroxyrifamycin S (1), 3-amino-rifamycin S (2), sporalactam A (3), sporalactam B (4), 27-demethoxy-27-hydroxyrifamycin S (5) and rifamycin S (6)	MRSA	MIC_90_ = 0.0008–7µM	Inhibition of transcription by binding to the β subunit (encoded by the *rpoB* gene) of the prokaryotic RNA polymerase (RNAP)	[131,132]
	-	*Nocardiopsis* sp. HB-J378 (sponge)	Nocardiopsistins A–C	MRSA	MIC = 3.12–12.5 μg/mL	-	[133]
	-	*Nonomuraea* sp. MM565M-173N2 (sediment)	Sealutomicins A–D (1–4)	MRSA (FDA 209P, MRSA No.5, MRSA No. 17, Mu50)	MIC = 0.0063–1.6 µg/mL	DNA scission via enediyne-generated biradicals (DNA cleavage) (3)	[134,135]
	-	*Paenibacillus profundus* sp. nov. SL 79 (sediment)	Isocoumarin	MSSA (CIP65.8T)	ZI = 46 mm (1 mg/mL)	-	[120]
	-	*Pseudomonas* sp. AMSN (algae)	2,4-Diacetylphloroglucinol (2,4 DAPG)	MRSA (ATCC43300)	MIC =8 µg/mL	Disruption of bacterial membrane integrity and inhibition of biofilm formation	[136,137]
	-	*Pseudomonas sp.* F92S91 (sponge)	Pyrone-I, II and III	GC 4541,4543: Smith,2216 MRSA (GC 1131)	MIC = 4–32 μg/mL		[138]
	-	*Shewanella algae* MTCC 12715 (red algae)	20-(20a,20b-dimethylbutan-20a-yl)-9-methoxy-3-methyl-dodecahydropyrano[3,4-*p*]-2,6,12-trioxacycloocta decine-1,13-dione (1) and Methyl-22-ethyl-5,6-dihydroxy-18-(hydroxymethyl)-4,5,9,22-tetramethyl-1,15,19-trioxo-octadecahydro-1H-benzo[*o*]-2,7,11,14-tetraoxacyclopentacosine-28-carboxylate (2)	MRSA (ATCC33592)	MIC = 3.12–5 µg/mL	-	[139]
	-	*Streptomyces althioticus* MSM3 (seaweed)	Desertomycin G	MSSA (ATCC25923) MRSA (ATCC 43300)	MIC = 4 µg/mL	-	[140]
	-	*Streptomyces cyaneofuscatus* M-169 (gorgonian coral)	Anthracimycin B	MSSA (ATCC 29213) MRSA (MB5393)	MIC = 0.125–8 µg/mL	-	[141]
	-	*Streptomyces koyangensis* SCSIO 5802 (sediment)	Neoabyssomicins F and G	MSSA (ATCC29213) MRSA (clinical isolates)	MIC = 16–32 µg/mL	-	[142]
	-	*Streptomyces lusitanus* OUCT16-27 (deep-sea sediment)	Grincamycin L (1), himalomycin B (2) and rabelomycin (3)	MRSA (CCARM 3090)	MIC = 6.25 (1) and >50 µg/mL (2 and 3)	-	[143]
	-	*Streptomyces platensis* TP-A0598 (seawater)	TPU-0037-A-D (30-demethyllydicamycin, 14, 15-dehydro-8-deoxylydicamycin)	209P JC-1 and MRSA (F597 and A2862)	MIC = 3.13–12.5 μg/mL	-	[144]
	-	*Streptomyces pratensis* strain NA-ZhouS1 (sediment)	Stremycins A and B	MRSA	MIC = 16 µg/mL	-	[145]
	-	*Streptomyces* sp. 12A35 (deep-sea sediment)	Lobophorins B, F, H and I, *O*-β-kijanosyl-(1→17)-kijanolide	MSSA (ATCC29213)	MIC = 6.25 to >100 µg/mL	-	[146]
	-	*Streptomyces* sp. 182SMLY (sediment)	*N*-acetyl-*N*-demethylmayamycin	MRSA (ATCC43300)	MIC = 20.0 μM	-	[147]
	-	*Streptomyces* sp. HZP-2216E (seaweed)	Bafilomycin D (1), 9-hydroxybafilomycin D (2), bafilomycin A1 (3) and 23-O-butyrylbafilomycin D (4)	MRSA	MIC = 7.4–32.2 µM	V-ATPase inhibitor (1)	[148]
	-	*Streptomyces* sp. HZP-2216E (seaweed)	21,22-en-bafilomycin D and 21,22-en-9-hydroxybafilomycin D	MRSA (ATCC43300)	MIC = 12.5 µg/mL	-	[85]
	-	*Streptomyces* sp. IMB7-145 (sediment)	Niphimycins C–E and Iα	MSSA (ATCC29213, isolates) MRSA (ATCC33591, isolates)	MIC = 8–64 µg/mL	-	[149]
	-	*Verrucosispora* sp. AB-18-032 (sediment)	Atrop-abyssomicin C	MRSA (isolates)	MIC = 20 µg/mL	Inhibition of para-aminobenzoic acid (pABA) biosynthesis, precursor of folic acid, via inhibition of 4-amino-4-deoxychorismate synthase	[150,151,152]
	-	*Verrucosispora* sp. AB-18-032 (sediment)	Abyssomicin C	MRSA (N315) MDR *S.aureus* (Mu50)	MIC = 4 (MRSA), 13 (MDR) µg/mL	Inhibition of para-aminobenzoic acid (pABA) biosynthesis, precursor of folic acid, via inhibition of 4-amino-4-deoxychorismate synthase	[153,154]
Terpenes	-	*Enterococcus lactis* (S-2) (sediment)	13-hydroxy-9-(1-hydroxyethyl)-11-methoxy-2,4-dioxapentacyclo [10.7.1.0^3^, ^4^.0^5^, ^21^.0^13^, ^16^] icosa-1(20),5,7,12,14(19),16-hexane-18-one	MSSA	MIC = 250 μg/mL	-	[155]
	-	*Micromonospora* sp. WMMC-218 (ascidian)	Micromonohalimane A	MRSA (ATCC33591)	MIC = 200 μg/mL	-	[156]
	-	*Micromonospora* sp. WMMC-218 (ascidian)	Micromonohalimane B	MRSA (ATCC33591)	MIC = 40 µg/mL	-	[156]
	-	*Streptomyces* sp. ZZ820 (coastal soil)	18-acetyl-cyclooctatin (1), 5,18-dedihydroxy-cyclooctatin (2), and 5-dehydroxy-cyclooctatin (3), cyclooctatin (4)	MRSA (ATCC43300)	MIC = 24.11–65.17 µg/mL	Potent inhibitor of lysophospholipase	[110,157]

### 2.3. Discussion on the Antibacterial Activity of Marine Bacterial Compounds Against S. aureus

**Figure 2:**
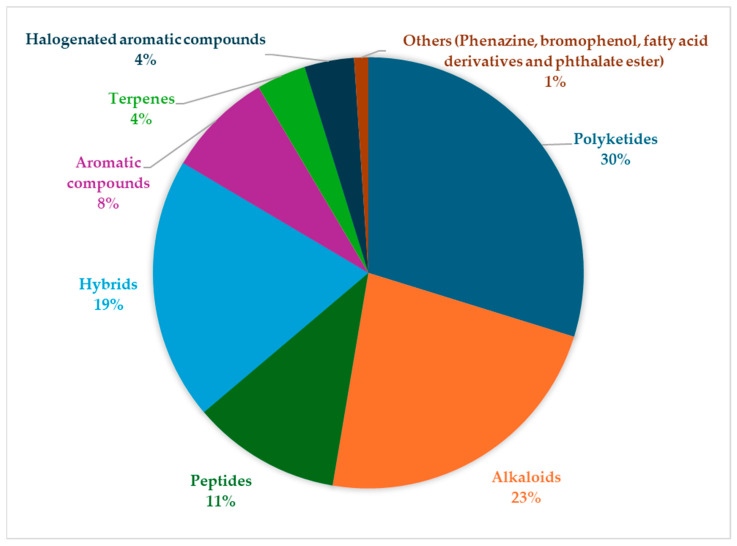
Distribution of bioactive compounds by chemical class. This diagram was created based on the data presented in Table 1. Each compound was individually classified according to its chemical class. The total number of identified compounds (*n* = 192) was then used to calculate the percentage representation of each category. This data was summarized in a diagram to illustrate the predominance of compound classes active against *S. aureus*.

The data compiled in Table 1 reveal a striking chemical diversity among marine bacterial compounds active against *S. aureus* strains. These compounds span several chemical families (see Figure 2), each with distinct structural motifs, activity spectra, and mechanisms of action. Below, we discuss these similarly grouped compounds, their strengths, weaknesses, and prospects for development.

#### 2.3.1. Polyketides

Polyketides constitute one of the most prominent and chemically diverse families of secondary metabolites produced by marine bacteria. These compounds are biosynthesized via modular polyketide synthases (PKSs), which allow for the assembly of complex carbon frameworks, often featuring multiple stereocenters, macrocycles, and functional group diversity.

Notable examples include: Abyssomicin C and its analogs, the neoabyssomicins, which exhibit Minimum Inhibitory Concentrations (MICs) generally ranging from 4 to 32 µg/mL against *S. aureus* strains [142,150]. Abyssomicin C acts as an inhibitor of para-aminobenzoic acid (pABA) biosynthesis, thereby disrupting folate-dependent metabolic pathways essential for bacterial survival [153].

Phocoenamicins and bafilomycins represent additional marine-derived polyketides, displaying a broader spectrum of MICs, depending on the bacterial strain tested [85,130]. The antibacterial mechanisms of bafilomycins are more diverse and often include interference with membrane functions or vacuolar-type ATPase inhibition [158].

These compounds illustrate both the therapeutic potential and inherent challenges associated with marine polyketides. While several demonstrate promising activity against *S. aureus*, including MRSA [49,159], their strain-dependent potency and biosynthetic complexity can hinder large-scale production and pharmacological optimization [160].

Moreover, their structural intricacy often complicates total synthesis and limits the feasibility of analog generation for structure–activity relationship (SAR) studies [161]. Nevertheless, polyketides remain a critical reservoir for novel antibacterial scaffolds, especially when coupled with genome mining and synthetic biology approaches aimed at enhancing yields and modulating bioactivity [162].

#### 2.3.2. Alkaloids

Alkaloids produced by marine bacteria represent a structurally diverse group of nitrogen-containing secondary metabolites with notable antibacterial activity, particularly against MRSA. Their activity often stems from unique mechanisms, including oxidative stress induction and interference with DNA.

Marinopyrroles A–F, isolated from *Streptomyces* sp. CNQ-418 is exemplary: they display potent activity (MIC_90_: 0.16–3.1 µg/mL) via membrane depolarization [83]. A prominent example is streptonigrin, isolated from *Streptomycetes* sp., which exhibits a potent MIC of approximately 0.78 µg/mL against MRSA. Its mode of action involves the generation of reactive oxygen species (ROS) and consequent DNA strand breakage, leading to rapid bacterial cell death [90].

The case of (E, Z)-albonoursin highlights the importance of stereochemistry in modulating antibacterial potency. This diketopiperazine alkaloid displays moderate activity, with MIC values of 12.5 µg/mL against MSSA and 25 µg/mL against MRSA, demonstrating how geometric isomerism can significantly influence biological outcomes.

Other structurally distinct marine alkaloids, including lynamicins, dionemycin, and vibripyrrolidines, exhibit a broad spectrum of activities, with MIC values ranging from 0.5 to 128 µg/mL. These variations underscore the critical role of substituent patterns, halogenation, and conformational flexibility in antibacterial efficacy [163]. Halogenation often enhances lipophilicity and membrane penetration, which may partially explain the higher potency observed in some halogenated analogs.

Overall, marine bacterial alkaloids combine potent antibacterial activity with structural novelty, making them compelling candidates for further lead optimization. Their amenability to chemical derivatization and their often non-classical modes of action also make them attractive for overcoming resistance mechanisms associated with traditional antibiotics.

#### 2.3.3. Peptides

Peptides, both ribosomal and non-ribosomal are synthesized and represent a highly potent class of marine-derived antibacterial agents. Their mechanisms of action often involve membrane disruption or inhibition of protein synthesis, which makes them particularly valuable in combating multidrug-resistant pathogens such as MRSA.

One of the most well-characterized examples is actinomycin D, a cyclic peptide exhibiting MIC values ranging from 0.125 to 1 µg/mL. Notably, this compound remains effective even against strains resistant to conventional antibiotics, owing to its ability to intercalate into DNA and inhibit RNA polymerase activity, thereby halting bacterial transcription and replication [164].

Taromycins A and B, isolated from *Saccharomonospora* sp., are lipopeptides structurally related to daptomycin, but with activity retained against daptomycin-resistant strains. Their MICs range from 3.1 to 50 µg/mL, indicating potential for further pharmacological development, particularly in resistant infection contexts where few options remain [121].

Similarly, fijimycins A–C and etamycin A act primarily by disrupting bacterial protein synthesis through ribosomal binding. However, their antibacterial activity is more variable, with MIC values reported between 4 and >32 µg/mL. This heterogeneity may stem from differences in uptake, stability, or ribosomal affinity, and suggests that SAR studies and chemical optimization would be necessary before clinical application.

Collectively, marine bacterial peptides offer a compelling avenue for antibiotic development. Their often-high potency, activity against resistant strains, and distinct mechanisms of action make them attractive leads. However, their stability, bioavailability, and potential cytotoxicity will require careful evaluation during preclinical development [165,166,167].

#### 2.3.4. Hybrids

Compounds that incorporate multiple structural motifs from distinct chemical families often display unique modes of action and stand out as particularly innovative scaffolds in the search for new antibiotics. These hybrid molecules frequently overcome the limitations of more conventional compound classes, offering new avenues for therapeutic intervention.

Among the most promising examples are the lipoxazolidinones A–C, a class of molecules featuring a hybrid oxazolidinone–peptide scaffold. These compounds exhibit MIC values ranging from 1 to 6 µg/mL against *S. aureus*, including methicillin-resistant strains. Their structural similarity to oxazolidinone antibiotics (e.g., linezolid) suggests a mechanism involving inhibition of protein synthesis, though further investigation is needed to fully elucidate their target spectrum [106,168].

Proximicin B, isolated from *Verrucosispora* sp., exemplifies another structurally innovative molecule. With a MIC of approximately 3.1 µg/mL, it integrates aromatic and peptide elements, potentially contributing to multi-target activity.

Similarly, the mindapyrroles and napyradiomycins exhibit highly variable antibacterial activity (MICs ranging from 0.5 to >96 µg/mL), strongly influenced by subtle structural variations, including halogenation patterns and oxidation states [92]. These findings underscore the importance of SAR analyses for optimizing potency and selectivity.

Perhaps the most potent compounds in this group are the chromomycins A_2_, A_3_, A_9_, and Ap, isolated from *Streptomyces microflavus*. These compounds show exceptionally low MIC values (0.06–0.25 µg/mL) against both MSSA and MRSA strains. Their mechanism involves direct inhibition of bacterial RNA polymerase, leading to suppression of transcription, a relatively underexploited target in antibiotic development [108,109,110]. This mode of action, distinct from that of traditional antibiotics, positions chromomycins as valuable candidates for overcoming resistance through novel target engagement.

Overall, hybrid and multi-motif compounds from marine bacteria highlight the chemical creativity of marine ecosystems. Their unconventional architectures and diverse biological targets support their prioritization in drug discovery pipelines, particularly as leads for resistant bacterial infections.

#### 2.3.5. Terpenes, Halogenated Aromatic Compounds, Aromatic Compounds and Minor Chemical Families: Potency in Rarity

Although underrepresented in number, terpenes, halogenated derivatives, and other structurally atypical marine metabolites contribute significantly to the antibacterial potential of marine bacterial secondary metabolites. These compounds often feature distinct biosynthetic origins, and in some cases, novel mechanisms of action that distinguish them from major chemical classes such as polyketides or peptides.

Among the terpenoids, compounds such as cyclooctatins and micromonohalimanes B, typically isolated from *Micromonospora* spp. and other rare marine Actinomycetes, exhibit moderate to weak activity, with MIC values generally ranging from 24 to >64 µg/mL against *S. aureus*. While their antibacterial potency is lower compared to other classes, these terpenes remain chemically intriguing and may serve as scaffold molecules for further optimization [169].

In contrast, halogenated compounds show significantly higher activity, benefiting from the electronic and lipophilic properties of halogen atoms, which enhance membrane permeability and target affinity. Notable examples include MC21-A and MC21-B, brominated phenolic compounds produced by *Pseudoalteromonas phenolica*, which display MIC values between 1 and 4 µg/mL against MRSA strains [95,100]. These compounds demonstrate how marine halometabolites can combine potency with relatively simple structures, offering favorable prospects for synthetic modification and pharmacological development.

Beyond these classes, several atypical metabolites exhibit remarkable antibacterial activity despite not fitting into traditional chemical categories. 7,8-Dideoxygriseorhodin C, a unique aromatic polyketide-like compound, stands out with MICs as low as 0.08–0.12 µg/mL, placing it among the most active molecules identified from marine bacteria. Other notable examples, such as phenazines, show MICs in the sub-micromolar to low micromolar range [113]. These molecules often act through underexplored mechanisms, such as oxidative stress induction, disruption of electron transport chains, or interference with bacterial redox homeostasis [170,171,172].

Collectively, these minor families emphasize the chemical breadth and mechanistic versatility of MNPs. Though fewer in number, they contribute disproportionately to the pool of high-potency candidates, reinforcing the importance of exploring non-canonical biosynthetic pathways in marine microbial communities [173,174].

#### 2.3.6. Spectrum of Activity Against *S. aureus* Strains

**Figure 3:**
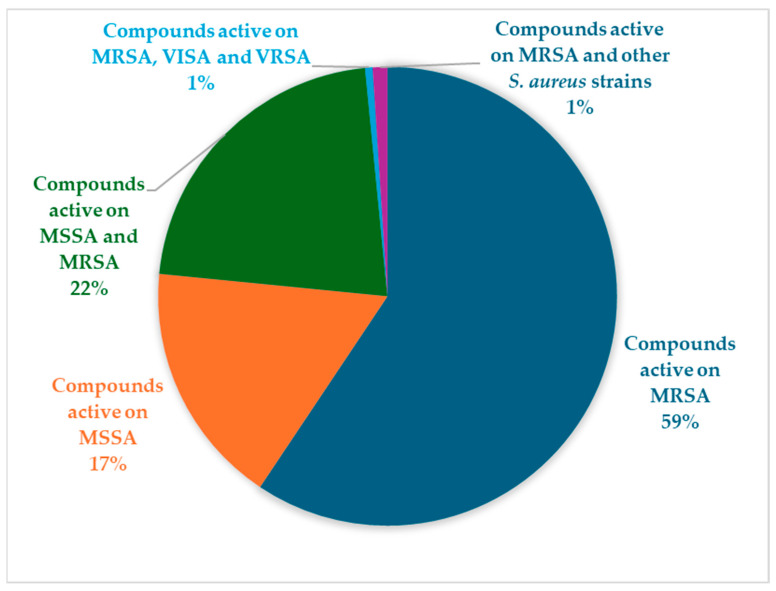
Distribution of compounds active against the *S. aureus* strains. This diagram was created based on the data presented in Table 1. Compounds targeting MSSA exclusively were counted separately from those active only against MRSA, while compounds active against both types of strains were grouped into a third category. Among the 192 compounds analyzed, the majority were active against MRSA alone, followed by a smaller proportion active only against MSSA, and a third group active against both. A limited number of compounds also showed broader activity, including those effective against MRSA, VISA, and VRSA, as well as compounds active against MRSA and other *S. aureus* strains not specifically classified as MSSA or resistant phenotypes. The percentage distribution for each category was calculated based on this total.

The antibacterial compounds isolated from marine bacteria predominantly target MRSA, reflecting the urgent clinical need to address antibiotic resistance (Figure 3). Most of the compounds have been tested either exclusively against MRSA or against both MRSA and MSSA strains, with only a smaller fraction showing activity solely against MSSA. This distribution underscores the strategic focus on developing agents effective against resistant pathogens.

The representation of the percentage distribution, 59% active against MRSA, 17% against MSSA, 22% against both, 1% against MRSA/VISA/VRSA, and 1% against MRSA and other *S. aureus* strains, provides valuable insight into the antibacterial spectrum of marine-derived compounds (see Figure 3). This pattern emphasizes a predominant activity toward MRSA, suggesting that MNPs may target resistance-associated mechanisms. The smaller proportion of compounds active against both MRSA and MSSA indicates the existence of broader-spectrum agents, whereas the rare activity against VISA and VRSA underscores the potential of marine metabolites to overcome even the most advanced resistance phenotypes. Moreover, since each *S. aureus* strain exhibits a distinct genomic profile influencing its virulence factors and resistance mechanisms, differences in antibacterial activity likely reflect underlying genetic diversity. Such quantitative representation facilitates the prioritization of promising candidates for further mechanistic studies and highlights the marine environment as a significant reservoir of bioactive molecules effective against multidrug-resistant pathogens.

**Figure 4:**
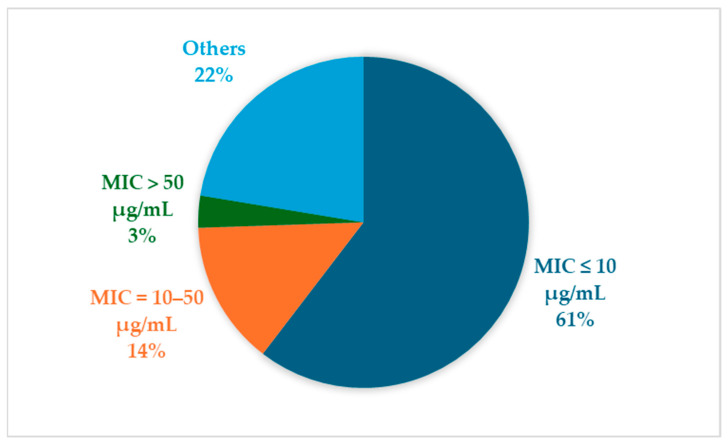
Classification of compounds by Minimum Inhibitory Concentration (µg/mL). This diagram was created based on the data presented in Table 1. MIC values were extracted for each compound from the dataset, focusing exclusively on those expressed in µg/mL. When MIC values were reported as a range, the lowest value was selected to reflect the most favorable antibacterial potential. Compounds were then categorized into three groups based on their minimum MIC ≤ 10 µg/mL (high activity), 10–50 µg/mL (moderate activity), and >50 µg/mL (low activity). Cases in which MIC data were missing, expressed in other units (e.g., µM and mm), or ambiguous data (e.g., MIC_90_, MIC_100_ and CI_50_) were grouped under “Others”. The percentage of compounds in each MIC range was then calculated relative to the total number of compounds considered (*n* = 192).

Regarding potency, approximately 61% of the studied compounds exhibit MICs below 10 µg/mL, classifying them as highly active and promising candidates for further drug development. Another 14% display moderate activity, with MICs between 10 and 50 µg/mL, warranting additional investigation into their pharmacodynamics and optimization potential. A smaller subset, about 3%, has MICs exceeding 50 µg/mL, indicating limited antibacterial efficacy in their current form; nonetheless, these molecules may serve as valuable leads for structural refinement. Although 22% of the compounds fell under the “Others” category due to missing or non-standardized MIC data, this subgroup represents a significant portion of the dataset and highlights the variability in data reporting across sources (see Figure 4).

Notably, several compounds demonstrate exceptional potency, achieving nanomolar MIC values in certain assays. Examples include inhibitors of bacterial chromosome replication and DNA-targeting agents, which highlight the potential of MNPs to yield powerful antimicrobials with novel mechanisms of action. Such high-affinity interactions further justify prioritizing these molecules for detailed mechanistic studies and preclinical evaluation (see Figure 4). This comprehensive distribution of activity and potency reflects the diverse chemical landscape of marine-derived antibacterial agents and provides a framework for rational prioritization in the search for new antibiotics effective against *S. aureus*, including MSSA and MRSA infections.

#### 2.3.7. Structural Trends Associated with Anti-*S. aureus* Activity

The compilation presented in Table 1 highlights the chemical diversity of antibacterial metabolites targeting *S. aureus* produced by marine bacteria, covering complex polyketides, alkaloids, peptides, terpenes, numerous structural hybrids, halogenated compounds, and other minor compound classes. However, beyond this quantitative richness, a cross-sectional analysis reveals that only a limited fraction of these compounds possesses characteristics compatible with realistic therapeutic development. A comparative analysis of the various chemical classes reveals the presence of several recurring structural motifs associated with anti-*S. aureus* activity, thereby substantiating structure–activity relationships (SARs).

Macrocyclic and highly constrained structures are often associated with low MICs. For instance, abyssomicin C targeting p-aminobenzoate biosynthesis, and anthracimycin B both show remarkable activity (MIC ranging from 0.125 to 4 µg/mL). Amphiphilic or membrane-disrupting motifs are also frequently observed in alkaloids and peptides active against MRSA. Marinopyrroles A–C and F, acting via the membrane depolarization and dissipation of the proton-motive force, are indeed highly active against MRSA (MIC_90_ = 0.16–3.1 µg/mL). Despite the fact that this mechanism is characterized by its rapid and potent activity, concerns regarding selectivity have been raised in relation to eukaryotic membranes [83]. Halogenation is also characteristic of marine metabolites. Halogenated biphenyls produced by *Pseudoalteromonas phenolica* (MIC = 1–4 µg/mL) or chlorinated bipyrrolic derivatives from *Streptomyces* sp. CNQ-418 (MIC_90_ = 0.78–6.3 µg/mL) illustrates how halogenation can enhance antibacterial potency. Moreover, halogenation agents have been studied for their potential role in combating bacterial resistance [175]. However, these same characteristics often constitute limiting factors: low metabolic stability, potential cytotoxicity, poor bioavailability, and synthetic complexity. Actinomycins are characterized by their extreme potency, as evidenced by their minimal inhibitory concentration (MIC) of less than 0.08 µM for actinomycin D. This property serves to illustrate a fundamental limitation in their utilization, namely the inseparability of their well-defined DNA intercalation mechanism from their high toxicity. Consequently, this restricts their application to the domain of oncology, as opposed to their use in antibiotic therapy [176]. The confrontation between high biological activity and chemical modification difficulties is a recurrent feature of marine metabolites effective against *S. aureus*.

Within the same chemical family, small structural modifications can also strongly modulate activity against *S. aureus*. For example, polyketides such as abyssomycins show that analogs with variations in the macrocycle or functional groups exhibit markedly different MICs, highlighting the importance of conformation and precise chemical features in enabling covalent interaction with the target enzyme involved in para-aminobenzoate biosynthesis (4-aminobenzoate), thereby blocking folate biosynthesis essential for bacterial growth [177]. In alkaloids, the marinopyrroles A–C and F possess halogen or hydroxyl substitutions that improve activity and selectivity compared to simpler analogues [83]. For peptides, fijimycins A–C and Etamycin A demonstrate that chain length or side-chain modifications directly impact the ability to inhibit bacterial protein synthesis [122]. Finally, among halogenated compounds, brominated or chlorinated biphenyls from *Pseudoalteromonas phenolica* illustrate how the presence and position of halogens influence antibacterial potency [95]. These examples underscore that structural optimization, even within a single chemical class, is crucial to maximize anti-*S. aureus* activity while minimizing toxicity and improving therapeutic potential.

Direct comparison of antibacterial activities reported in the literature must be interpreted with caution. Indeed, biological results are expressed as MIC, MIC_90_, MIC_100_, IC_50_, or inhibition zones, coming from heterogeneous experimental protocols (Table 1). Moreover, highlighting compounds solely based on low MICs, without cytotoxicity or stability data, remains insufficient. Sealutomicins A–D illustrate this ambiguity: despite extremely low MICs (up to 0.0063 µg/mL), their mechanism, based on enediyne biradical generation causing DNA breaks, raises major toxicity and safety concerns. Conversely, compounds such as lipoxazolidinones or lynamicins exhibit more moderate potency but a structure more compatible with chemical optimization, meaning that their chemical framework is easier to modify to improve pharmacological properties, reduce toxicity, and enhance drug-like characteristics without losing antibacterial activity [79,168]. However, most marine bacteria-derived compounds have been tested mainly against cancer cell lines [178,179]. Thus, antimicrobial assays, including MIC values, should be considered indicative of antibacterial potential, not as direct predictors of clinical value.

#### 2.3.8. Challenges and Future Perspectives

Despite the promising antibacterial activity demonstrated by marine bacterial metabolites, several challenges must be addressed to advance these compounds toward clinical application.

First, comprehensive data on pharmacokinetics, toxicity, and bioavailability remain scarce for many molecules. Notably, some compounds exhibit significantly reduced efficacy in the presence of human serum, as observed with marinopyrrole A, limiting their systemic therapeutic potential [83]. Toxicity and innocuity are also major limitations for translational studies. For instance, the polyketides sealutomicins exert high anti-MRSA activity with low MIC values but raise major safety concerns as they possibly cause DNA breaks [134]. Addressing these limitations through medicinal chemistry approaches, such as the development of structural analogs to improve solubility, stability, serum compatibility, and molecule safety by decreasing toxicity, is essential.

Second, production and synthetic accessibility pose significant hurdles. The structural complexity inherent to many marine natural products often complicates large-scale synthesis or fermentation. To overcome this, strategies including total or semi-synthesis, heterologous expression of biosynthetic gene clusters, and synthetic biology approaches are promising avenues to ensure sustainable and scalable compound supply.

Third, the modes of action (MOA) of a substantial portion of these metabolites remain poorly characterized. Integration of cutting-edge omics techniques, genomics, transcriptomics, and metabolomics, combined with biophysical and structural biology methods, will be critical to elucidate their molecular targets. Such insights are invaluable for optimizing efficacy and circumventing resistance mechanisms.

Lastly, marine bacterial compounds often exhibit anti-virulence properties, including inhibition of biofilm formation and disruption of *Quorum-Sensing* pathways. These mechanisms provide complementary therapeutic strategies by attenuating pathogenicity rather than solely killing bacteria, potentially reducing the selective pressure for resistance development.

Addressing these challenges through multidisciplinary efforts will accelerate the translation of marine-derived antibacterial compounds into effective therapeutics against *S. aureus* and other resistant pathogens.

Marine bacterial metabolites represent a highly valuable and under-exploited source of structurally diverse antibacterial agents against *S. aureus*, especially MRSA strains. Correct classification of compounds into their proper chemical families enhances our understanding of their biosynthetic origins, mechanisms, and development challenges. With focused efforts on optimizing the most potent compounds, elucidating mechanisms of action, and overcoming production and pharmacokinetic hurdles, many of these marine natural products stand poised to contribute to future antibiotic therapies.

## 3. Marine-Derived Antibiotics: Advances, Bottlenecks, and Prospects in the Exploration of Marine Bacteria

### 3.1. Standard Methods for the Isolation and Identification of Antibacterial Metabolites

**Figure 5:**
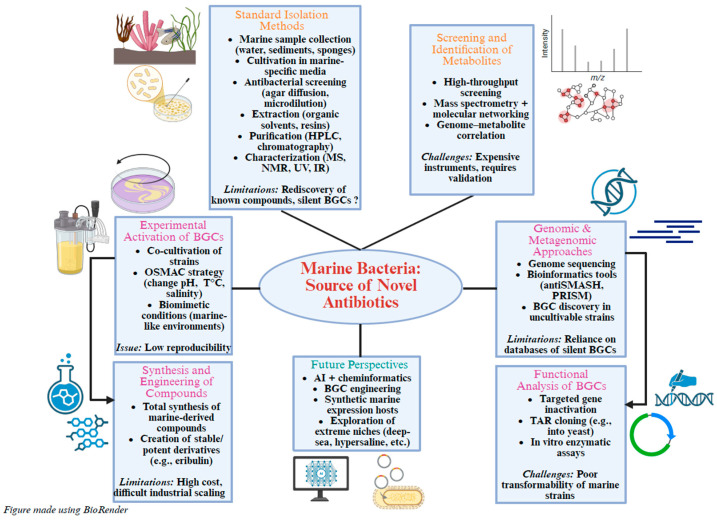
Overview of classical and emerging methods for antibiotic discovery from marine bacteria; Schematic overview illustrating the different stages of antibiotic discovery from marine bacteria, from classical approaches (in orange), such as isolation, screening, fermentation, extraction, and structural characterization of metabolites, to emerging approaches (in purple), including genomics, activation of biosynthetic gene clusters, functional analyses, high-throughput screening, metabolomics, and chemical synthesis. Abbreviations: HPLC: High-Performance Liquid Chromatography; MS: Mass Spectrometry; NMR: Nuclear Magnetic Resonance; UV: Ultraviolet Spectroscopy; IR: Infrared Spectroscopy; BGCs: Biosynthetic Gene Clusters; OSMAC: One Strain–Many Compounds; AI: Artificial Intelligence; TAR: Transformation-Associated Recombination. Created with BioRender.com. C, Xuma (2026).

Isolation and identification of antibacterial metabolites produced by marine bacteria require a series of steps combining microbiological, chemical, and molecular methods (see Figure 5). The process begins with the collection of marine samples, such as seawater, sediments, or marine organisms like sponges or corals, followed by bacterial isolation using culture media tailored to marine conditions, such as Zobell medium or Marine Broth 2216E. The isolated strains are then screened for antibacterial activity using techniques like agar diffusion, direct confrontation, or liquid microdilution assays to detect inhibition of the growth of target pathogenic bacteria [180]. While often overlooked, geographical and environmental diversity in marine sampling strongly affects chemical diversity; exploring extreme habitats may uncover many undescribed compounds [53]. Although this step is particularly time-consuming and labor-intensive, it remains essential due to the lack of effective alternative methods, ensuring the selection of the most promising strains for further analysis. Active strains are then cultured under fermentation conditions to promote the production of secondary metabolites, which are extracted using organic solvents (such as methanol or ethyl acetate) or by adsorption onto resins.

The crude extracts are then fractionated and purified using various chromatographic techniques, including column chromatography, High-Performance Liquid Chromatography (HPLC), or gas chromatography for volatile compounds. Each fraction is tested for antibacterial activity to validate bioactivity and identify the bioactive components. Once isolated, these metabolites are characterized using advanced analytical chemistry methods, notably mass spectrometry (MS), nuclear magnetic resonance (NMR), ultraviolet–visible (UV-Vis) spectroscopy, and infrared (IR) spectroscopy, to elucidate their chemical structures. The utilization of databases, such as NPAtlas and LOTUS [181], can facilitate the early detection of structural redundancy, thereby minimizing the effort expended on the rediscovery of previously identified compounds.

These approaches have led to significant discoveries. Around 28,000 MNPs are currently known, and more than 1000 new compounds have been isolated each year over the past decade [182,183]. For example, marinomycin A, derived from *Marinispora* spp., was identified through classical isolation techniques and showed strong activity against multidrug-resistant bacteria such as *S. aureus* [184]. However, these methods present important limitations. First, they frequently lead to the rediscovery of already known compounds [185], which slows down chemical innovation and reduces the pharmaceutical value of new isolations. In addition, the initial culture-based methods may fail to recover certain bacterial isolates, especially slow-growing or fastidious strains, further limiting the diversity of metabolites accessible for screening. Second, they rely on the natural expression of metabolites under standard laboratory culture conditions, which prevents the detection of many compounds produced by silent or poorly expressed gene clusters. These constraints now justify the integration of complementary strategies such as data mining, including genome mining, and heterologous expression to fully exploit the potential of marine bacteria.

### 3.2. Genomic, Metagenomic, and In Silico Approaches for the Exploration of BGCs

Approaches such as genome mining, genomics, metagenomics, in silico analyses, and omics-based methods (see Figure 5), made possible through the recent improvements of bioinformatics during the last decades, allow the exploration of the biosynthetic potential of microorganisms, especially marine microbes, by identifying biosynthetic gene clusters (BGCs) and predicting their roles in the production of secondary metabolites [59,186]. These techniques include the analysis of biosynthetic enzymes, bioinformatic comparisons, and simulated or actual heterologous expression in model hosts [187]. Tools such as antiSMASH or PRISM [188,189], for example, enable the annotation of PKS/NRPS-type BGCs in the genome of marine bacteria or in environmental samples that are difficult to culture, through metagenomic approaches. One major application has been the discovery of new BGCs in marine Actinomycetes associated with sponges, through metagenomic sequencing combined with in silico annotation [190]. Importantly, this type of approach also allows researchers to leverage sequences obtained for other applications, meaning that data generated for one study can be reanalyzed or repurposed to explore additional BGCs, thereby maximizing the utility of existing genomic and metagenomic datasets. However, these approaches present two main limitations: first, the frequent lack of expression of the identified BGCs, which often remain silent under standard laboratory conditions [191], and second, their reliance on reference databases, which may limit the accuracy of predictions when dealing with truly novel or atypical BGCs.

### 3.3. Functional Analysis of Biosynthetic Pathways

Functional approaches aim to elucidate how identified BGCs are organized and regulated, and how they lead to the production of secondary metabolites (see Figure 5). These approaches encompass several experimental methods: targeted gene inactivation allows for the deletion of specific genes to observe the consequent loss of the associated metabolite production; phylogenetic analyses assist in predicting enzyme functions; synthetic substrates can be introduced to confirm enzymatic activity *in vitro* [192]. More advanced tools, such as Transformation-Associated Recombination (TAR) cloning [193] enable the capture of large BGCs into hosts like yeast, while plasmid sharing [194] and overexpression techniques [195] facilitate enhanced production of target compounds. These methods have been successfully applied, for example, to validate the biosynthesis of novel marine molecules in Actinomycetes [196]. However, challenges remain, including the fact that many marine strains are genetically difficult to manipulate, as they are often resistant to genetic transformation, and the intrinsic complexity of many biosynthetic pathways, which often require highly specific host systems for successful expression [197]. To overcome this, Gao et al. (2019) developed integrative vectors based on TG1 and R4 integrases and successfully achieved conjugative transfer from *E. coli* to the Actinomycete *Amycolatopsis marina*, demonstrating both the challenge and potential solutions to working with such strains [197]. This example highlights the technical barriers often faced when exploring marine microbial diversity and the need for tailored genetic tools.

### 3.4. Experimental Methods for Expression and Activation of BGCs

A significant proportion of BGCs identified through genome mining remain silent under laboratory conditions (see Figure 5). To activate these clusters, various experimental strategies have been developed [191]. Co-cultivation [198,199,200,201], which involves growing two microorganisms together, can induce competitive or synergistic interactions that trigger the expression of silent genes. The OSMAC (One Strain, Many Compounds) approach relies on modifying culture parameters such as temperature, salinity, pH, and nutrient composition to influence metabolic expression [202,203,204]. Furthermore, biomimetic strategies [205,206] aim to replicate the natural environment, such as extreme marine conditions, to awaken silent biosynthetic pathways. These methodologies have enabled the discovery of several bioactive compounds from marine bacteria previously considered low producers. However, these approaches often suffer from low reproducibility and variable efficacy depending on the strain, complicating their standardization for high-throughput screening.

### 3.5. High-Throughput Screening, Analysis, and Identification Techniques for Bioactive Compounds

Once BGCs are activated, it is crucial to detect and characterize the compounds they produce. High-throughput screening [207] allows for the rapid testing of numerous microbial extracts to identify biological activities (see Figure 5). In parallel, mass spectrometry coupled with molecular networking analysis [208] enables visualization of relationships between structurally similar compounds by clustering them based on chemical similarity. When these data are combined with genome mining results [209], it becomes possible to link a detected metabolite to a specific biosynthetic gene cluster, thereby facilitating its annotation. This integrated approach has enabled the rapid identification of entire families of metabolites in marine bacteria. However, these techniques require expensive analytical infrastructure, and genome–metabolite correlations often remain hypothetical without further validation through compound purification and targeted biological assays.

### 3.6. Synthesis and Engineering of Bioactive Compounds

Total synthesis is a strategy used to chemically recreate natural compounds, particularly those of marine origin, which are often produced in very low quantities (see Figure 5). This approach allows for confirmation of a metabolite’s structure, the production of more stable or potent derivatives, and the development of optimized versions for therapeutic use. A notable example is the synthesis of eribulin [210], a simplified analog of halichondrin B, originally isolated from a marine sponge [211], and now approved for use in oncology [212]. Despite its advantages, total synthesis is often time-consuming, expensive, and technically challenging, especially when it comes to scaling up production for industrial applications.

### 3.7. Critical Evaluation, Limitations, and Perspectives

Despite the range of available approaches, the discovery of new antibiotics from marine bacteria still faces several major challenges. A significant proportion of BGCs identified through comparative genomics or metagenomics are redundant, often leading to the rediscovery of already known metabolites. This highlights the urgent need to re-evaluate certain “forgotten” or poorly characterized molecules, which may reveal novel biological activities if studied in alternative therapeutic contexts. Indeed, a single compound may exhibit unexpected pharmacological profiles depending on the infectious model being targeted. Moreover, although current bioinformatics tools are powerful, they still lack the precision needed to reliably predict the chemical structure or biological activity of a metabolite solely based on its genetic signature. Coupling artificial intelligence with cheminformatics may eventually improve these predictive capabilities [213,214,215,216] (see Figure 5).

Technical limitations also represent a significant barrier to the valorization of marine-derived compounds into viable therapeutic solutions. These include low production yields, chemical instability, the difficulty of compound isolation at scale, and the lack of compatible heterologous expression systems. As a result, even when promising molecules are identified, the path from discovery to clinical application can take over a decade. For example, *eribulin*, a synthetic analog of halichondrin B (originally isolated from the marine sponge *Halichondria okadai*), took more than 20 years from its initial discovery in the late 1980s to FDA approval in 2010 for the treatment of metastatic breast cancer [211,217,218]. Similarly, the development of salinosporamide A (Marizomib), derived from the marine Actinomycete *Salinispora tropica*, required extensive optimization and has only recently entered late-stage clinical trials for glioblastoma, nearly 15 years after its first report [219,220]. Moreover, to the best of our knowledge, no bacterial MNP has been approved for antibacterial application yet.

These cases illustrate that the transition from laboratory discovery to a marketable drug involves complex and time-consuming steps, including preclinical validation, toxicological assessment, formulation, manufacturing scale-up, and regulatory approval. To overcome these bottlenecks, new strategies are emerging: BGC bioengineering [221,222,223], the use of synthetic host strains optimized for marine gene expression [222,224], ultra-high-throughput screening assisted by relevant cellular models [225,226,227,228,229,230], and the exploration of extreme ecological niches (e.g., hydrothermal vents, hypersaline environments) that remain largely untapped (see Figure 5).

By integrating these multidisciplinary approaches and streamlining the development pipeline, marine bioprospecting may reach a new level of productivity and contribute significantly to the future antimicrobial arsenal, particularly against multidrug-resistant pathogens such as *S. aureus*.

## 4. Conclusions

The continued emergence of multidrug-resistant *S. aureus*, including MRSA, VISA, and VRSA, highlights the pathogen’s exceptional adaptability and the growing limitations of conventional antibiotics. Beyond its resistant forms, *S. aureus* remains one of the most clinically significant and versatile bacterial pathogens, responsible for a wide range of infections in both community and healthcare settings. This underscores the urgent need for innovative therapeutic strategies targeting *S. aureus*. In this critical context, MNPs produced by marine bacteria represent a promising, yet still underexploited, source of secondary metabolites with both antibacterial and anti-virulence activities. The adaptation of these microorganisms to extreme marine environments has driven exceptional biosynthetic diversity, often surpassing that of terrestrial microbes. Recent advances in marine microbiology, genomics, metagenomics, and biotechnology now enable more effective exploration of this resource through genome mining, heterologous expression, and high-throughput screening. The overall analysis of this review suggests that the most promising marine chemical space against *S. aureus* does not necessarily lie in the most complex or *in vitro* potent structures, but in moderately complex, chemically modifiable scaffolds associated with clearly identified mechanisms of action. Optimizable peptides, for instance, bogorol A and fijimycins, some rationalizable hybrids, such as lipoxazolidinones, and simple halogenated compounds appear as priority candidates. Conversely, extremely complex macrocycles and metabolites with highly genotoxic mechanisms, while chemically fascinating, present major barriers to therapeutic exploitation. Nevertheless, important challenges persist, including chemical redundancy, difficulties in activating silent BGCs, low yields of key compounds, and the intrinsic complexity of translating marine-derived molecules into therapeutic agents. Despite these hurdles, recent technological progress provides encouraging prospects. The integration of classical microbiological methods with innovative biotechnological approaches, alongside a deeper understanding of marine microbial ecology, could unlock this latent potential. Fully incorporating marine bacteria into antibiotic discovery pipelines represents not only a realistic strategy to counteract *S. aureus*, but also a critical step in the broader fight against antimicrobial resistance.

## Data Availability

No new data was created or analyzed in this study. Data sharing is not applicable to this article.

## Abbreviations

**antiSMASH**	Antibiotics & Secondary Metabolite Analysis Shell
**BGC**	Biosynthetic Gene Cluster
**DNA**	Deoxyribonucleic Acid
**EtOAc**	Ethyl Acetate
**FDA**	Food and Drug Administration
**GC**	Gas Chromatography
**HGT**	Horizontal Gene Transfer
**HPLC**	High-Performance Liquid Chromatography
**HTS**	High-Throughput Screening
**IR**	Infrared (Spectroscopy)
**LC-MS**	Liquid Chromatography–Mass Spectrometry
**MeOH**	Methanol
**MDR**	Multidrug-Resistant
**MNPs**	Marine Natural Products
**MS**	Mass Spectrometry
**NGS**	Next-Generation Sequencing
**NMR**	Nuclear Magnetic Resonance
**NRPS**	Non-Ribosomal Peptide Synthetase
**OMICS**	Comprehensive biological analysis approaches (genOMIC, transcriptOMIC, proteOMIC, metabolOMIC)
**OSMACs**	One Strain, Many Compounds
**PCR**	Polymerase Chain Reaction
**PKS**	Polyketide Synthase
**PRISM**	Prediction Informatics for Secondary Metabolomes
**TAR**	Transformation-Associated Recombination
**UV-Vis**	Ultraviolet–Visible (Spectroscopy)
**WT**	Wild Type
